# Clinopyroxene precursors to amphibole sponge in arc crust

**DOI:** 10.1038/ncomms5329

**Published:** 2014-07-08

**Authors:** Daniel J. Smith

**Affiliations:** 1 Department of Geology, University of Leicester, University Road, Leicester LE1 7RH, UK

## Abstract

The formation of amphibole cumulates beneath arc volcanoes is a key control on magma geochemistry, and generates a hydrous lower crust. Despite being widely inferred from trace element geochemistry as a major lower crustal phase, amphibole is neither abundant nor common as a phenocryst phase in arc lavas and erupted pyroclasts, prompting some authors to refer to it as a ‘cryptic’ fractionating phase. This study provides evidence that amphibole develops by evolved melts overprinting earlier clinopyroxene—a near-ubiquitous mineral in arc magmas. Reaction-replacement of clinopyroxene ultimately forms granoblastic amphibole lithologies. Reaction-replacement amphiboles have more primitive trace element chemistry (for example, lower concentrations of incompatible Pb) than amphibole phenocrysts, but still have chemistries suitable for producing La/Yb and Dy/Yb ‘amphibole sponge’ signatures. Amphibole can fractionate cryptically as reactions between melt and mush in lower crustal ‘hot zones’ produce amphibole-rich assemblages, without significant nucleation and growth of amphibole phenocrysts.

## Abstract (web summary)

Studies have suggested amphibole to be a major phase in the lower crust based on trace element geochemistry of volcanic suites, although the formation of these lithologies remains cryptic. Here, the author suggests a reaction-replacement mechanism to explain how this cryptic fractionation may occur.

## Graphical abstract

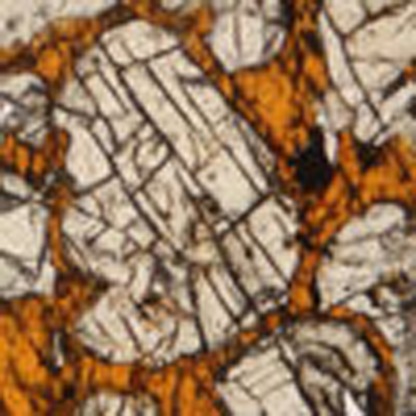

The composition of the lower crust of arcs plays an important role in magmatism and mantle-to-upper crust fluxes of metals and volatiles^1^,^2^. Nominally anhydrous, clinopyroxene-dominated lithologies in the lower crust are refractory and prone to foundering^3^,^4^. In comparison, amphibole-bearing assemblages are more prone to melting^5^,^6^,^7^,^8^ and/or assimilation^9^,^10^. However, the fractionation of amphibole is often considered to be cryptic^1^,^11^,^12^ given the lack of amphibole phenocrysts in many arc lavas and pyroclasts^13^, even where the presence of amphibole cumulates and autoliths is unequivocal^14^. Conversely, clinopyroxenites are less common as cumulates and autoliths than fractionation models suggest, particularly in mature arc crust^15^.

Savo, Solomon Islands, southwest Pacific (Fig. 1), is a recently eruptive volcano formed by the on-going subduction of the Indo-Australian plate beneath the Solomon Islands Arc^16^. Savo Island is the upper third of the 1,400-m high edifice of the volcano, which is built onto an unknown basement^17^. Crustal thickness is ~14 km (ref. 17). The mantle beneath Savo, and much of the Solomon Islands Arc, has potentially been metasomatized by slab melts from the earlier-subducted Pacific slab, and the currently subducting Indo-Australian slab^18^,^19^,^20^,^21^,^22^. Historical eruptions (16th and 19th centuries^17^) have been dominated by mugearites to sodic trachytes (mildly alkaline equivalents of basaltic andesite to dacite)^18^. The interpreted fractionation sequence is typical of arc magmas with early oxides and olivine followed closely by clinopyroxene, with later amphibole^18^. Plagioclase feldspar is a ubiquitous phenocryst phase in the erupted volcanic suite, but with limited involvement in the major geochemical evolution of the suite, and is interpreted to crystallize late (that is, upon or just before extrusion of the magma) with little to no participation in fractionation, leading to elevated Sr/Y values (100–250 in the evolved rocks)^17^. Amphibole occurs as a phenocryst in the more evolved parts of the suite, but consistent La/Yb and Dy/Yb trends across the range of analysed SiO_2_ indicate its fractionation from more primitive melts too—hence fractionation is cryptic early, and non-cryptic later in the suite.

This study examines the suite at Savo volcano, in particular the phenocrysts and entrained nodules of hornblendites, clinopyroxenites and mixed amphibole–clinopyroxene lithologies. Nodules are interpreted to represent a range of cumulates and reaction-replacement lithologies, with considerable amphibole formed by melt–clinopyroxene reactions. Consistent with experimental literature and empirical evidence from a range of amphibole-bearing lithologies, the first appearance of amphibole in crystallizing (mafic) arc magma is likely to be at the expense of clinopyroxene (and/or olivine) by reaction-replacement. This reaction-replacement mechanism is effective at generating a ‘cryptic’ fractionation signature for amphibole as it develops in relatively immobile crystal mushes, and unlike true cumulates, there need not be crystal phases nucleating and growing in the liquid melt. Amphibole phenocrysts at Savo have greater enrichments of incompatible trace elements relative to the nodules, suggesting their separate and later crystallization from more evolved melts. The decoupling of phenocrysts and nodule chemistry illustrates that crystal fractionation may be cryptic, in that early formation and fractionation of minerals is not necessarily recorded in the crystal assemblage of the daughter magmas, and that phenocryst population is not representative of the crystallization history of a magma.

## Results

### Mineralogy and petrography

**Figure 2:**
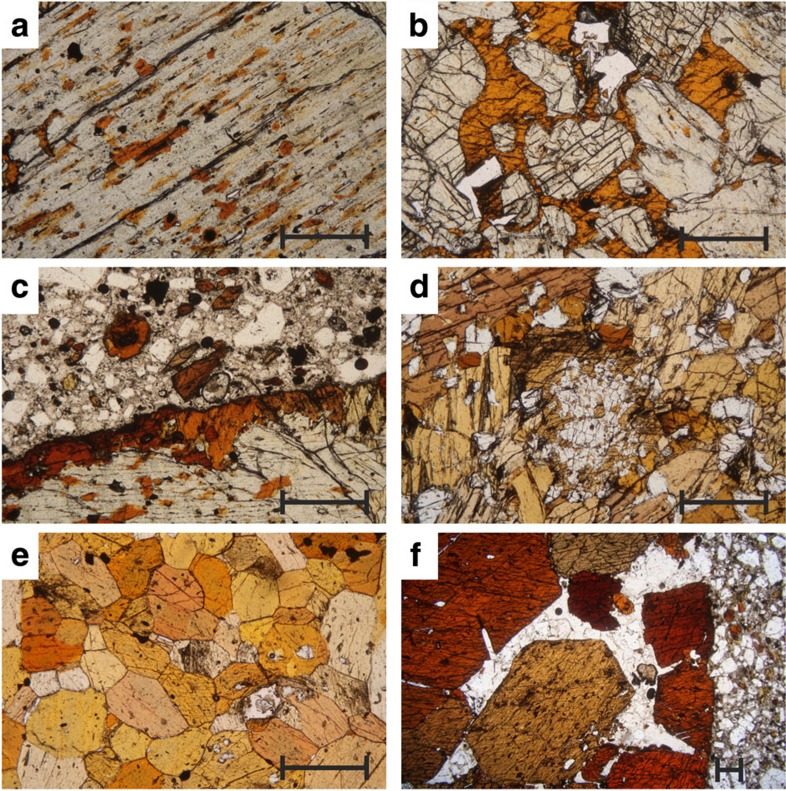
Thin section photomicrographs of cumulate nodules from Savo. (**a**) Orange amphibole blebs developing along cleavage planes of a clinopyroxene in a clinopyroxenite nodule. (**b**) Orange ‘poikilitic’ amphibole replacing clinopyroxene, preserved as rounded kernels with the amphibole. (**c**) Brown amphibole rim at contact between clinopyroxenite nodule and amphibole-phyric trachyte. (**d**) Relict clinopyroxene in a hornblendite. (**e**) Granoblastic hornblendite. (**f**) Idiomorphic amphibole cumulate (with inter-cumulate apatite and calcic plagioclase) in an amphibole-phyric trachyte. The scale bars in the lower right of each image are 0.5 mm across.

The erupted rocks of Savo contain abundant nodules (Fig. 2) of clinopyroxenites (±olivine), hornblendites and assemblages with varying proportions of clinopyroxene and amphibole (±olivine). Gabbroic assemblages are rare, with plagioclase occurring rarely as a highly calcic, anhedral phase. This is in line with the chemistry of the evolved rocks (Eu anomaly poorly developed or absent, Sr behaves as an incompatible element), which suggests a limited role for plagioclase fractionation^18^, likely a result of elevated H_2_O in the magma^4^,^23^. Magnetite is present in all samples as a minor phase, often as inclusions, suggesting it is an early-crystallizing mineral.

Clinopyroxenites are dominated (90% by volume) by clinopyroxenes that straddle the augite-diopside composition. Olivine occurs as isolated crystals within the nodules, and is typically Fo_70–80_. Clinopyroxenites contain variable amounts of amphibole, and can be considered an end member of a continuum through mixed amphibole–clinopyroxene to hornblendites (Fig. 2a–e).

All amphiboles are hornblende group (*sensu lato*) and are dominantly pargasites and hornblendes. The majority of hornblendites (±clinopyroxene) display granoblastic textures with subhedral amphibole (Fig. 2e). A much smaller number of samples contain idiomorphic amphibole (no clinopyroxene) in an anhedral groundmass of calcic plagioclase and apatite (Fig. 2f).

Nodules were analysed for whole rock geochemistry by X-ray fluorescence (major and trace elements) and solution inductively coupled plasma mass spectrometry (trace elements), and crystal chemistry by scanning electron microscope–energy dispersive X-ray microanalysis (SEM-EDXA; major elements) and laser ablation inductively coupled plasma mass spectrometry (LA-ICP-MS; trace elements). Details of analyses are described in Methods and Supplementary Data 1.

## Discussion

The textures of amphibole in clinopyroxenites and clinopyroxene–amphibole cumulates through to granoblastic hornblendites support a reaction-replacement^24^ origin, with replacement of clinopyroxene as blebs along cleavage planes^25^ (Fig. 2a) at crystal margins (producing pseudo-interstitial and poikilitic textures; Fig. 2b) and at nodule rims (Fig. 2c). Ultimately reaction replacement produces hornblendite with a granoblastic texture, and varying proportions of subhedral, relict clinopyroxene (Fig. 2d,e). The formation of amphibole by melt–clinopyroxene reaction is a widely reported and long-recognized phenomenon in cumulates^26^,^27^, mantle pyroxenites^28^ and metasomatized peridotitic mantle^29^,^30^. Idiomorphic, framework adcumulates (Fig. 2f) are precipitation cumulates, formed by the nucleation, growth and settling of amphibole from a melt^24^.

**Figure 3:**
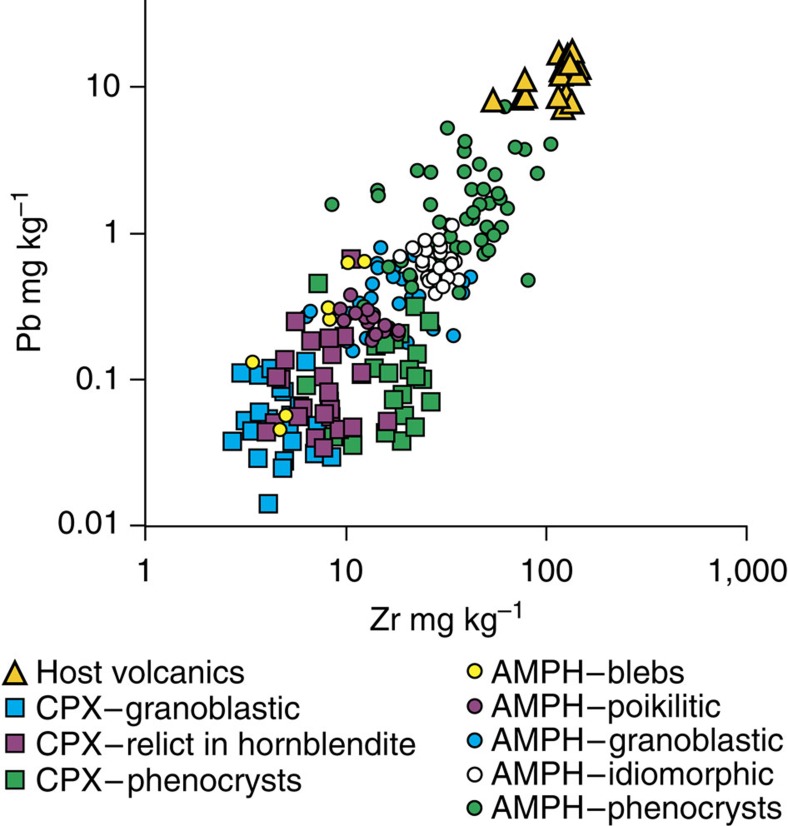
Trace element chemistry of Savo amphibole and clinopyroxene organized into mineral and textural groups. Phenocrysts of a given mineral generally show greater enrichments of the incompatible trace elements than cumulate and reaction-replacement equivalents, suggesting phenocrysts crystallize from more evolved melt. Amphiboles show increasing enrichment in the textural sequence: bleb–poikilitic–granoblastic–idiomorphic–phenocryst. Crystal data by LA-ICP-MS. Whole rock data by X-ray fluorescence data (from ref. 17). Typical 1σ error is within point size for both data sets.

The trace element geochemistry (see Supplementary Data 1) of the samples can be used to determine a genetic sequence for these textural classes. Incompatible elements such as Zr and Pb increase in the melt with continued fractionation; later phases should have higher concentrations of these elements. Figure 3 shows that the mineral–textural sequence: clinopyroxene–amphibole bleb–crystal margins–granoblastic amphibole is reflected in the trace element geochemistry. Clinopyroxene phenocrysts are generally enriched in incompatible trace elements (average values in mg kg^−1^: Zr 18, Pb 0.15, Th 0.03 and Rb 0.08) compared with clinopyroxene in cumulates (Zr 5, Pb 0.06, Th 0.01 and Rb 0.05); similarly, amphibole phenocrysts are enriched in incompatibles (Zr 41, Pb 1.7, Th 0.11 and Rb 2.6) compared with the reaction-replacement amphiboles (Zr 18, Pb 0.38, Th 0.04 and Rb 1.2). Idiomorphic amphibole cumulates are equivalent to the least evolved phenocrysts (Zr 28, Pb 0.65, Th 0.03 and Rb 1.7). Decoupling of phenocryst and cumulate populations indicates that cumulates form earlier than phenocrysts, and that populations of the latter may not be representative of the former.

**Figure 4:**
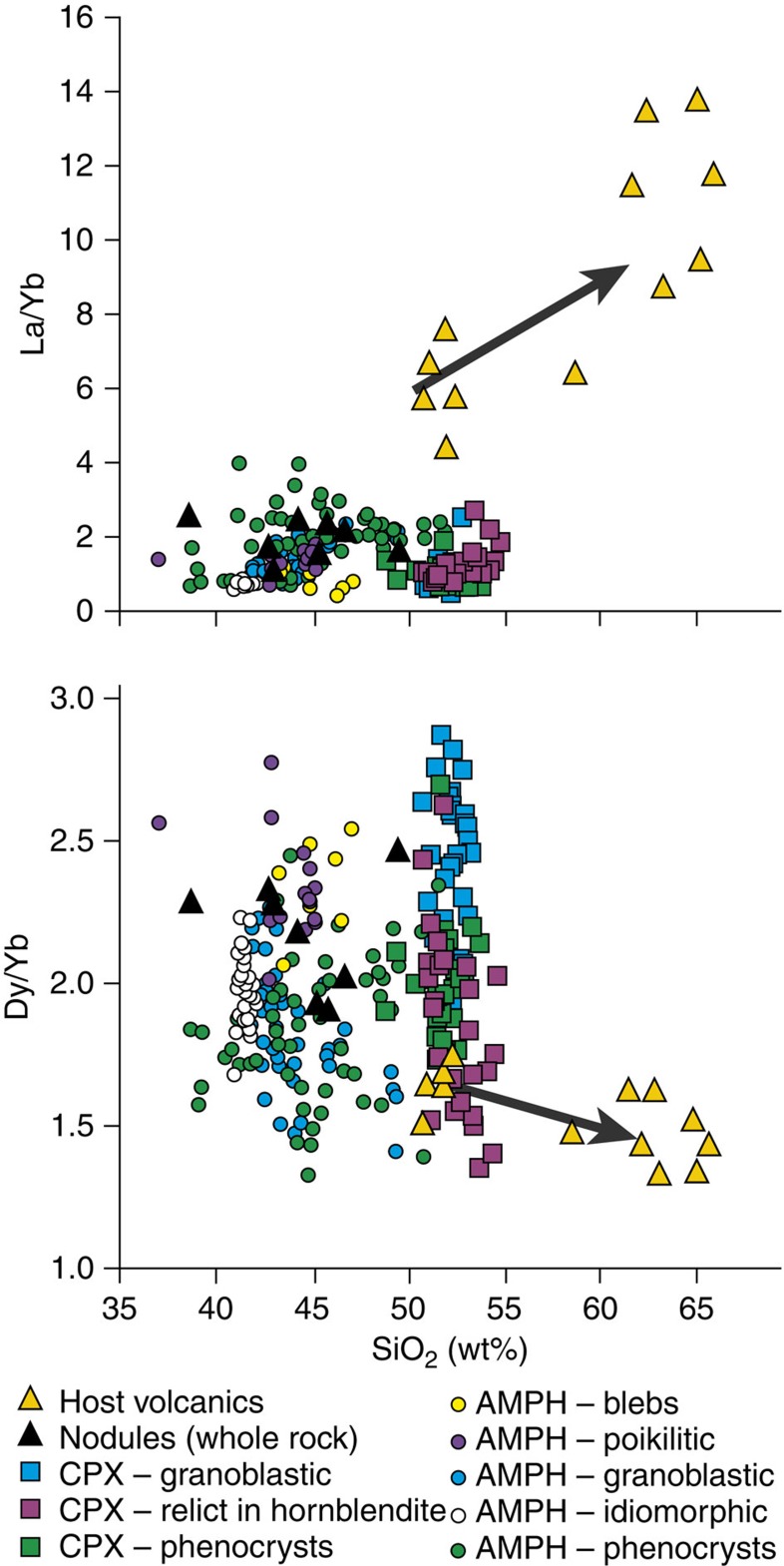
Indicators of amphibole fractionation. La/Yb and DyYb versus SiO_2_ for host volcanics^18^, whole rock nodules (this study; solution ICP-MS) and mineral analyses (this study; LA-ICP-MS). Arrows mark typical amphibole fractionation vectors^1^, which the host volcanics approximately follow.

**Figure 5:**
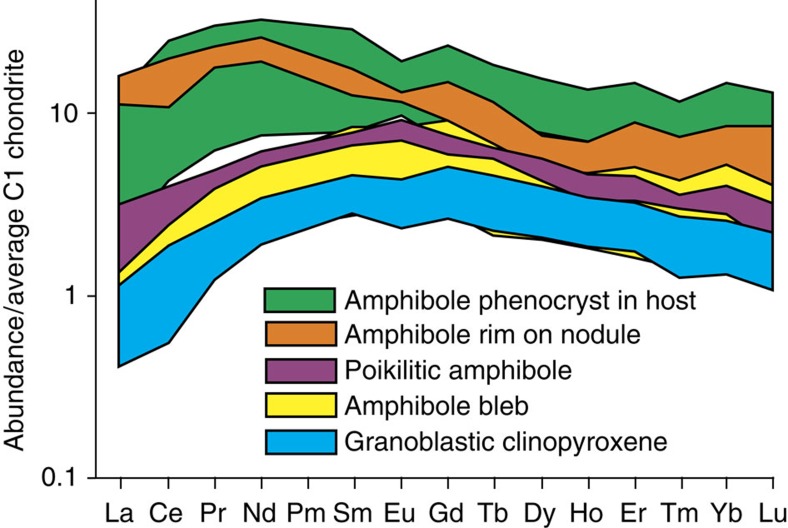
Rare earth element profiles. Chondrite-normalized^42^ values shown for selected crystals from clinopyroxenite nodule with reaction-replacement amphibole in trachyte host (sample SV158; shown in Fig. 2c). Amphibole bleb and precursor clinopyroxene have very similar REE profiles; there is a progressive enrichment in the REE with texturally later amphibole (poikilitic and nodule rim). The amphibole rim of the nodule is chemically similar to the host rock phenocrysts.

Despite the implied variation in parent melt, and the different formative processes of amphibole, all populations are capable of contributing to an ‘amphibole sponge’ La/Yb and Dy/Yb signature (Fig. 4). Normalized rare earth element (REE) profiles (see Fig. 5) show similar forms—albeit at different relative enrichments—for clinopyroxenes and amphiboles of different texture classes.

Volcanic suites with La/Yb, Dy/Yb trends indicative of amphibole fractionation (including Savo; Fig. 4) show no inflections marking an ‘amphibole-in’ point in the petrogenetic sequence, and the suites show amphibole fractionation patterns from the most mafic parts to the most evolved^1^. Dy/Dy* trends in arc magmas also suggest amphibole as a key fractionating phase, but do not preclude clinopyroxene involvement^31^. Experimental data on hydrous basaltic fractionation^32^,^33^,^34^, and the maximum thermal stability of amphibole (~1,100 °C; ref. 35) both support the formation of clinopyroxene before amphibole, which, as in this study, develops at the expense of the earlier-formed clinopyroxene (and/or olivine). Progression from clinopyroxene to amphibole fractionation is not marked by inflections in La/Yb and Dy/Yb because: (a) REE ratios for clinopyroxene and hornblende are similar; (b) clinopyroxene fractionation alone has little impact on daughter melt SiO_2_ (ref. 1) and hence, trends in SiO_2_ space are suppressed; and (c) this study demonstrates that amphibole fractionation may be partial and progressive, which will smooth any abrupt changes in chemistry inferred from a single ‘mineral-in’ phase boundary. The continuous trend in the Savo La/Yb, Dy/Yb data from ~50 wt% SiO_2_ (whole rock), as well as those of Davidson *et al.*^1^, coupled with the relatively limited influence of clinopyroxene on residual melt SiO_2_, means that the melts participating in amphibole-forming melt–mush reactions are expected to be low SiO_2_ (basalt or basaltic andesite), with relatively low abundances of incompatible trace elements, albeit higher than the melt before clinopyroxene crystallization (reflected in the crystal chemistry; Fig. 3).

Melts reacting with clinopyroxene need not be co-genetic; clinopyroxene may be formed as a cumulate from earlier magmas, and progressively replaced with amphibole by later melts ascending through the cumulate pile. The formation of an ‘amphibole sponge’ in the lower crust of arcs can therefore occur by repeated injection of melts into, and reactive transport^36^ through hot zone-type systems^37^. The lower thermal stability of amphibole means that these cumulates may be periodically re-melted by the addition of high-temperature (>1,110 °C) primitive melts, providing a mechanism by which lower crustal sulphides^3^ entrained within cumulate assemblages are reworked into ascending magmas and ultimately contribute to metallogenesis.

Amphibole-forming reactive transport of a melt through a clinopyroxene mush will impart an amphibole fractionation signature^36^ irrespective of amphibole appearance or absence as a phenocryst phase. The absence of amphibole phenocrysts in melts segregated from the mush may be explained by a range of non-exclusive mechanisms. The precursory clinopyroxene mush may already have surpassed the liquid-to-solid transition^38^ and be effectively immobilized in the lower crust.

**Figure 6:**
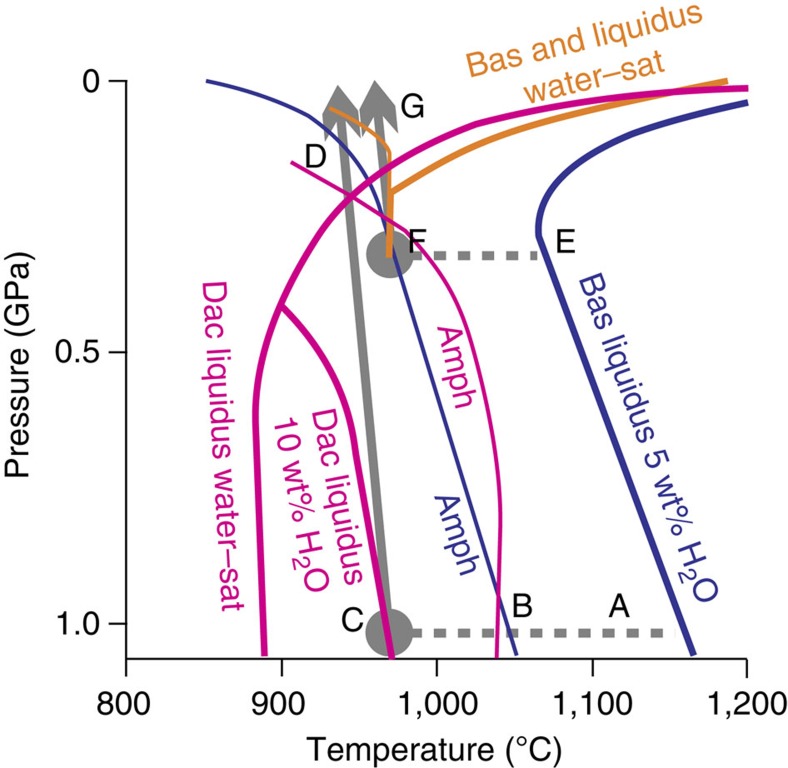
Composite pressure–temperature phase diagram for arc magmas. This shows the liquidus (heavy lines, liquid to the right of each line) and amphibole-in boundary (fine lines; amphibole to the left of each line) for basalt with 5 w% H_2_O (ref. 32) (blue), water-saturated basaltic andesite^43^ (orange) and dacite^37^ (pink; water saturated and 10 wt% H_2_O). Grey arrows show adiabatic magma ascent^37^ from crustal hot zones. Dashed lines show cooling–crystallizing paths of basaltic melt injected into the hot zone. Path A–D shows a 25-km-deep continental hot zone forming a dacitic melt^37^ and E–G a basaltic andesite from a 14-km-deep hot zone.

Figure 6 shows mechanisms by which reaction-replacement may act to generate cryptic fractionation of amphibole. Path A–D shows a basaltic melt injected into a 25-km-deep crustal hot zone^37^. As basalt cools and crystallizes along path A–B, clinopyroxene and olivine are the dominant silicate phases. At B, amphibole appears at the expense of clinopyroxene^32^. At C, a dacitic liquid with 10 wt% H_2_O is extracted from the hot zone and ascends adiabatically. Along C–D, the liquid is super-liquidus, and any entrained crystals may be resorbed^37^; this crustal interval will therefore be underrepresented or absent in the crystal record of the magma. At point D, the dacitic melt crosses the liquidus and begins to crystallize, but outside of the amphibole stability field. Hence, the magma has been subject to fractionation at 1.0 GPa (equivalent to the lower crust of a continental arc), with no phenocryst record of amphibole. Path E–F illustrates basalt injection, cooling and crystallization at an initial pressure of 0.3 GPa (comparable to the lower crust of an intra-oceanic arc, and the setting for Savo). Fractionation is olivine and clinopyroxene dominated, with amphibole appearing at the expense of clinopyroxene at F. Basaltic andesite liquids (similar to the most primitive melts bearing amphibole phenocrysts erupted at Savo) extracted from the melt–mush pile at F, ascend adiabatically along F–G. With reference to the basaltic andesite phase fields, the extracted liquid can quickly intersect the liquidus and crystallize amphibole during ascent until relatively low pressures. Thus, the magmas erupted from point G may carry non-resorbed cumulates from F, and will be amphibole-phyric, although phenocrysts may show decompression-related breakdown due to moving out of the stability field of amphibole in the shallow crust^39^. The high Na_2_O content of the Savo magmas^18^ may increase the stability field of amphibole at lower pressures^23^.

The onset of amphibole formation in primitive arc magmas is at the expense of clinopyroxene precursors. Where intermediate and more evolved arc magmas are generated at hot zones, amphibole fractionation can be achieved by residual melts reacting with earlier-formed clinopyroxene mushes. Melt segregation in hot zones^38^ provides mechanisms by which a largely crystal-free melt is separated from the early-formed clinopyroxene and reaction-replacement amphiboles. Phenocryst formation is decoupled from this process, and as a result, fractionation of amphibole in arc magmas may be cryptic.

## Methods

### Whole rock chemistry

Whole rock cumulate samples were analysed with a PANalytical Axios Advanced XRF spectrometer at the University of Leicester. Major element analyses determined on fused glass beads prepared from ignited powders using a 1:5 sample to (80% Li metaborate:20% Li tetraborate) flux ratio. Trace elements were determined from 32 mm diameter pressed powder pellets produced by mixing 7 g fine ground sample powder with 12–15 drops of a 7% polyvinyl alcohol solution (Moviol 8–88). A range of reference materials were used to calibrate both instruments. The precision (1σ) of the major element data, across a range of compositions, was estimated to be <3% for SiO_2_, Al_2_O_3_, Fe_2_O_3_, MgO, CaO and Na_2_O, <7% for TiO_2_ and MnO, and <10% for K_2_O and P_2_O_5_. For trace elements at concentrations above 10 p.p.m. the precision was <15% for Ba; <10% for Ce, Co, Cr, Cu, Ni, Sc; and <5% for Ga, Rb, Sr, V, Zn, Zr. For trace elements at concentrations below 10 p.p.m., the estimated for precision were <1 p.p.m. for Ga, Nb, Rb; <2 p.p.m. for Co, Cu, La, Nd, Ni, Y; and <4 p.p.m. for Ba, Cr, Th, Zr. Measured values for reference materials were within 1σ of accepted values.

Additional trace element on whole rock cumulate samples were analysed by solution ICP MS at Durham University, using a Thermo Scientific X-Series 2 ICP-MS, and digestion methods outlined in ref. 40 Statistical errors and detection limits are included in Supplementary Data 1.

### Microanalytical chemistry

Microanalytical major element data are from the British Geological Survey’s FEI QUANTA 600 environmental SEM, coupled with an Oxford Instruments INCA Energy 450 EDXA system that utilizes an Oxford Instruments X-Max large area (50 mm^2^) Peltier-cooled silicon-drift detector. Quantitative EDXA analyses were carried out using an accelerating voltage of 20 kV. Reproducibility for the major elements is ±2% of the quoted values, based on repeat spot analysis.

Microanalytical trace element data are by LA-ICP-MS, with a New Wave Research UP-213 laser ablation system with He carrier gas, attached to a Thermo Finnigan Element 2 ICP-MS at the University of Notre Dame’s Midwest Isotope and Trace Element Research Analytical Center (MITERAC). The laser was operated at 5 Hz with a 5-μm spot size, with analysis intervals of ~30 s background counts, ~20 s laser warm-up, 60 s ablation and ~45 s washout. Elemental abundances were determined using GLITTER software (XP version, Simon Jackson, Macquarie University) with Ca from EDXA-SEM used as the internal standard and NIST SRM 612 glass used as the external standard. Statistical errors and detection limits are included in Supplementary Data 1.

## Author contributions

D.J.S. designed the study, performed the analyses and wrote the paper.

## Additional information

**How to cite this article:** Smith, D. J. Clinopyroxene precursors to amphibole sponge in arc crust. *Nat. Commun.* 5:4329 doi: 10.1038/ncomms5329 (2014).

## Supplementary Material

Supplementary Data 1 Microanalytical and whole rock geochemical data of nodules (clinopyroxenites, hornblendites and amphibole-pyroxene lithologies) from Savo, Solomon Islands.

## Figures and Tables

**Figure 1:**
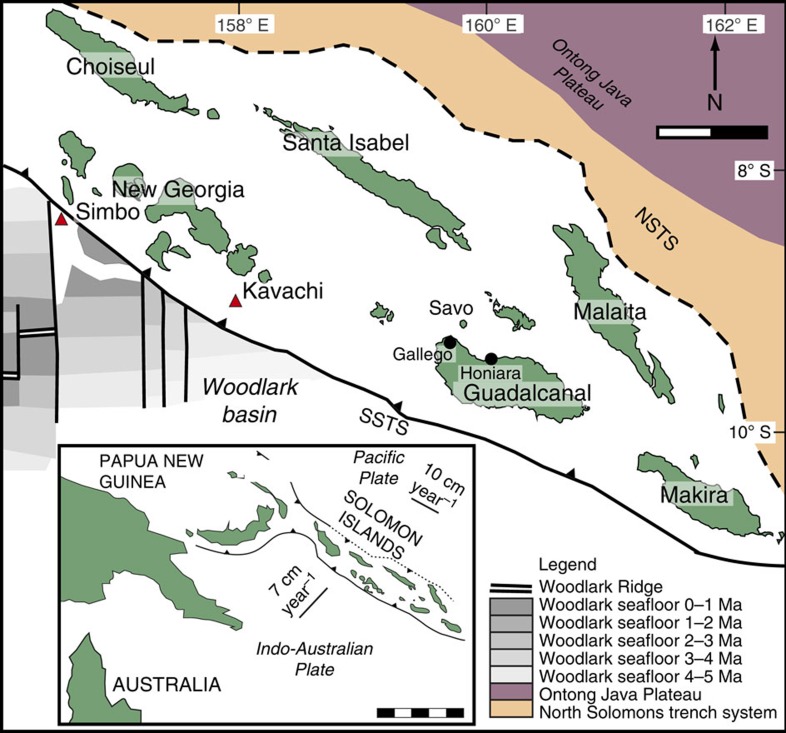
Map of the Solomon Islands. Savo Island, major tectonic features and Kavachi and Simbo volcanoes are labelled. Woodlark seafloor ages from ref. 41. Scale bar, 100 km at 50-km intervals. Inset shows major plate boundaries of the southwest Pacific, with plate motions from ref. 16. Inset scale bar, 500 km at 100-km intervals.

## References

[b1] Davidson J., Turner S., Handley H., Macpherson C. & Dosseto A. Amphibole sponge in arc crust? Geology 35, 787–790 (2007).

[b2] Richards J. P. Postsubduction porphyry Cu-Au and epithermal Au deposits: Products of remelting of subduction-modified lithosphere. Geology 37, 247–250 (2009).

[b3] Lee C.-T. A. *et al.* Copper systematics in arc magmas and implications for crust-mantle differentiation. Science 336, 64–68 (2012). 22491850 10.1126/science.1217313

[b4] Müntener O., Kelemen P. & Grove T. The role of H_2_O during crystallization of primitive arc magmas under uppermost mantle conditions and genesis of igneous pyroxenites: an experimental study. Contrib. Mineral. Petrol. 141, 643–658 (2001).

[b5] Beard J. S. & Lofgren G. E. Dehydration melting and water-saturated melting of basaltic and andesitic greenstones and amphibolites at 1, 3, and 6. 9 kb. J. Petrol. 32, 365–401 (1991).

[b6] Brown M. The generation, segregation, ascent and emplacement of granite magma: the migmatite-to-crustally-derived granite connection in thickened orogens. Earth Sci. Rev. 36, 83–130 (1994).

[b7] Sorbadere F., Schiano P. & Metrich N. Constraints on the origin of nepheline-normative primitive magmas in island arcs Inferred from olivine-hosted melt inclusion compositions. J. Petrol. 54, 215–233 (2012).

[b8] Médard E., Schmidt M. W., Schiano P. & Ottolini L. Melting of amphibole-bearing wehrlites: an experimental study on the origin of ultra-calcic nepheline-normative melts. J. Petrol. 47, 481–504 (2006).

[b9] Dungan M. A. & Davidson J. Partial assimilative recycling of the mafic plutonic roots of arc volcanoes: An example from the Chilean Andes. Geology 32, 773–776 (2004).

[b10] Tiepolo M., Langone A., Morishita T. & Yuhara M. On the recycling of amphibole-rich ultramafic intrusive rocks in the arc crust: evidence from Shikanoshima Island (Kyushu, Japan). J. Petrol. 53, 1255–1285 (2012).

[b11] Kratzmann D. J., Carey S., Scasso R. A. & Naranjo J. A. Role of cryptic amphibole crystallization in magma differentiation at Hudson volcano, Southern Volcanic Zone, Chile. Contrib. Mineral. Petrol. 159, 237–264 (2010).

[b12] Larocque J. & Canil D. The role of amphibole in the evolution of arc magmas and crust: the case from the Jurassic Bonanza arc section, Vancouver Island, Canada. Contrib. Mineral. Petrol. 159, 475–492 (2010).

[b13] Ewart A. Mineralogy and chemistry of modern orogenic lavas — some statistics and implications. Earth Planet. Sci. Lett. 31, 417–432 (1976).

[b14] Arculus R. J. & Wills K. J. A. The petrology of plutonic blocks and Inclusions from the Lesser Antilles Island Arc. J. Petrol. 21, 743–799 (1980).

[b15] Sorbadere F., Médard E., Laporte D. & Schiano P. Experimental melting of hydrous peridotite–pyroxenite mixed sources: Constraints on the genesis of silica-undersaturated magmas beneath volcanic arcs. Earth Planet. Sci. Lett. 384, 42–56 (2013).

[b16] Petterson M. G. *et al.* Geological-tectonic framework of Solomon Islands, SW Pacific; crustal accretion and growth within an intra-oceanic setting. Tectonophysics 301, 35–60 (1999).

[b17] Petterson M. G. *et al.* The eruptive history and volcanic hazards of Savo, Solomon Islands. Bull. Volcanol. 65, 165–181 (2003).

[b18] Smith D. J. *et al.* The petrogenesis of sodic island arc magmas at Savo volcano, Solomon Islands. Contrib. Mineral. Petrol. 158, 785–801 (2009).

[b19] Schuth S. *et al.* Petrogenesis of lavas along the Solomon Island Arc, SW Pacific: Coupling of compositional variations and subduction zone geometry. J. Petrol. 50, 781–811 (2009).

[b20] König S., Schuth S., Münker C. & Qopoto C. The role of slab melting in the petrogenesis of high-Mg andesites: evidence from Simbo Volcano, Solomon Islands. Contrib. Mineral. Petrol. 153, 85–103 (2007).

[b21] König S. & Schuth S. Deep melting of old subducted oceanic crust recorded by superchondritic Nb/Ta in modern island arc lavas. Earth Planet. Sci. Lett. 301, 265–274 (2011).

[b22] Petterson M. G., Haldane M. I., Smith D. J., Billy D. & Jordan N. J. Geochemistry and petrogenesis of the Gallego Volcanic Field, Solomon Islands, SW Pacific and geotectonic implications. Lithos 125, 915–927 (2011).

[b23] Sisson T. W. & Grove T. L. Experimental investigations of the role of H_2_O in calc-alkaline differentiation and subduction zone magmatism. Contrib. Mineral. Petrol. 113, 143–166 (1993).

[b24] Irvine T. N. Terminology for layered intrusions. J. Petrol. 23, 127–162 (1982).

[b25] Veblen D. R. & Buseck P. R. Hydrous pyriboles and sheet silicates in pyroxenes and uralites: intergrowth microstructures and reaction mechanisms. Am. Mineral. 66, 1107–1134 (1981).

[b26] Best M. G. Amphibole–bearing cumulate inclusions, Grand Canyon, Arizona and their bearing on silica-undersaturated hydrous magmas in the upper mantle. J. Petrol. 16, 212–236 (1975).

[b27] Debari S., Kay S. M. & Kay R. W. Ultramafic Xenoliths from Adagdak Volcano, Adak, Aleutian Islands, Alaska: Deformed Igneous Cumulates from the Moho of an Island Arc. J. Geol. 95, 329–341 (1987).

[b28] Francis D. Amphibole pyroxenite xenoliths: Cumulate or replacement phenomena from the upper mantle, Nunivak Island, Alaska. Contrib. Mineral. Petrol. 58, 51–61 (1976).

[b29] Coltorti M. *et al.* Amphibole genesis via metasomatic reaction with clinopyroxene in mantle xenoliths from Victoria Land, Antarctica. Lithos 75, 115–139 (2004).

[b30] Neal C. R. The origin and composition of metasomatic fluids and amphiboles beneath Malaita, Solomon Islands. J. Petrol. 29, 149–179 (1988).

[b31] Dessimoz M., Muntener O. & Ulmer P. A case for hornblende dominated fractionation of arc magmas: the Chelan Complex (Washington Cascades). Contrib. Mineral. Petrol. 163, 567–589 (2012).

[b32] Foden J. D. & Green D. H. Possible role of amphibole in the origin of andesite: some experimental and natural evidence. Contrib. Mineral. Petrol. 109, 479–493 (1992).

[b33] Cawthorn R. G. & O'Hara M. J. Amphibole fractionation in calc-alkaline magma genesis. Am. J. Sci 276, 309–329 (1976).

[b34] Melekhova E., Annen C. & Blundy J. Compositional gaps in igneous rock suites controlled by magma system heat and water content. Nat. Geosci. 6, 385–390 (2013).

[b35] Ridolfi F. & Renzulli A. Calcic amphiboles in calc-alkaline and alkaline magmas: thermobarometric and chemometric empirical equations valid up to 1,130°C and 2.2 GPa. Contrib. Mineral. Petrol. 163, 877–895 (2012).

[b36] Reiners P. W. Reactive melt transport in the mantle and geochemical signatures of mantle-derived magmas. J. Petrol. 39, 1039–1061 (1998).

[b37] Annen C., Blundy J. D. & Sparks R. S. J. The genesis of intermediate and silicic magmas in deep crustal hot zones. J. Petrol. 47, 505–539 (2006).

[b38] Solano J. M. S., Jackson M. D., Sparks R. S. J., Blundy J. D. & Annen C. Melt segregation in deep crustal hot zones: a mechanism for chemical differentiation, crustal assimilation and the formation of evolved magmas. J. Petrol. 53, 1999–2026 (2012).

[b39] Rutherford M. J. & Hill P. M. Magma ascent rates from amphibole breakdown: An experimental study applied to the 1980–1986 Mount St. Helens eruptions. J. Geophys. Res. B: Solid Earth 98, 19667–19685 (1993).

[b40] Ottley C. J., Pearson D. G. & Irvine G. J. inPlasma Source Mass Spectrometry: Applications and Emerging Technologies eds Holland J. G., Tanner S. D. 221–230Royal Society of Chemistry (2003).

[b41] Taylor B. inMarine Geology, Geophysics and Geochemistry of the Woodlark Basin-Solomon Islands eds Taylor B., Exon N. F. 25–48Circum-Pacific Council for Energy and Mineral Resources (1987).

[b42] Anders E. & Ebihara M. Solar-system abundances of the elements. Geochim. Cosmochim. Acta 46, 2363–2380 (1982).

[b43] Moore G. & Carmichael I. S. E. The hydrous phase equilibria (to 3 kbar) of an andesite and basaltic andesite from western Mexico: constraints on water content and conditions of phenocryst growth. Contrib. Mineral. Petrol. 130, 304–319 (1998).

